# Efficient Integration of Synaptic Events by NMDA Receptors in Three-Dimensional Neuropil

**DOI:** 10.1016/j.bpj.2015.04.009

**Published:** 2015-05-19

**Authors:** Kaiyu Zheng, Dmitri A. Rusakov

**Affiliations:** 1 UCL Institute of Neurology, University College London, Queen Square, London, United Kingdom

## Abstract

Sustained activation of NMDA receptors (NMDARs) plays an important role in controlling activity of neural circuits in the brain. However, whether this activation reflects the ambient level of excitatory neurotransmitter glutamate in brain tissue or whether it depends mainly on local synaptic discharges remains poorly understood. To shed light on the underlying biophysics here we developed and explored a detailed Monte Carlo model of a realistic three-dimensional neuropil fragment containing 54 excitatory synapses. To trace individual molecules and their individual receptor interactions on this scale, we have designed and implemented a dedicated computer cluster and the appropriate software environment. Our simulations have suggested that sparse synaptic discharges are 20–30 times more efficient than nonsynaptic (stationary, leaky) supply of glutamate in controlling sustained NMDAR occupancy in the brain. This mechanism could explain how the brain circuits provide substantial background activation of NMDARs while maintaining a negligible ambient glutamate level in the extracellular space. Thus the background NMDAR occupancy, rather than the background glutamate level, is likely to reflect the ongoing activity in local excitatory networks.

## Introduction

High-affinity NMDA receptors (NMDARs) play a critical role in signal transfer and use-dependent synaptic plasticity in the brain. In addition to their classical contribution to rapid responses at excitatory synapses, in quiescent brain tissue NMDARs appear to mediate a persistent 50–200 pA current in individual pyramidal cells (revealed by removing the receptor Mg^2+^ block) (1, 2, 3, 4). Correspondingly, extensive evidence in vivo points to a prominent role of sustained NMDAR activation in controlling excitability of various neuronal circuits (5, 6, 7, 8, 9). However, whether this activation is mediated predominantly by synaptic discharges or by the constituent presence (or leakage) of glutamate in the extracellular space is poorly understood.

Careful measurements of the ambient glutamate concentration in quiescent acute hippocampal slices give 25–50 nM (4, 10), or 3–6 molecules per 1 *μ*m^3^ of neuropil, given the extracellular space fraction of ∼0.2 (11). This exceptionally low level is generally consistent with the concentration equilibrium due to the stoichiometry of high-affinity glutamate transporters (12) expressed in abundance by hippocampal astroglia (13), and is not due to transmitter washout in the acute slice preparation (4). The main source of this glutamate appears to involve sustained nonsynaptic release (3, 4), probably from astroglia (3). The NMDAR kinetics (14, 15) predict that this low steady-state glutamate concentration activates only <0.1% of the NMDAR population. The latter estimate agrees with the tonic 50–200 pA NMDAR current detected in slices (see above) given a one-receptor current of ∼1 pA and 10,000–20,000 NMDARs per hippocampal pyramidal cell (16, 17, 18).

At first glance, ambient glutamate supplied through nonsynaptic leakage is thus a plausible candidate for sustained NMDAR in vivo: indeed, it has been suggested that a typical hotspot of glutamate generated by individual synaptic discharges has a half-life of only <1 ms and an effective spatial domain of <0.5 *μ*m (19, 20, 21, 22, 23). Previous studies also estimated that extrasynaptic glutamate escape upon a single release event at an individual synapse is limited by high-affinity glutamate transporters, typically allowing only a small proportion of extrasynaptic NMDARs (occurring within ∼0.5 *μ*m from the release site) to singly-bind escaped glutamate molecules (20, 23). Similarly, steady-state release, such as nonsynaptic leakage, also must overcome powerful glutamate uptake in order to elevate activation of NMDARs beyond a very low ambient level, as indicated above. An alternative suggestion has therefore been that NMDARs could serve as a powerful space- and time-integration device for rapid, relatively sparse discharges of glutamate from activated synapses (24). Furthermore, in hippocampal neuropil, the intrasynaptic density of NMDARs is two orders-of-magnitude higher than that outside synapses (16, 17). Thus, even though the postsynaptic densities (areas enriched in synaptic receptors) of excitatory synapses occupy only 1–2% of the interstitial space (25), the total numbers of intra- and extrasynaptic NMDARs appear comparable. This puts into perspective the relative physiological impact of nonsynaptic glutamate actions. Nonetheless, which of the two mechanisms—individual synaptic discharges or continued leakage of glutamate—could more efficiently sustain continued NMDAR activation in organized brain tissue, remains poorly understood.

To obtain quantitative insights into this issue, we explored a Monte Carlo model that traces individual glutamate molecules in a 3-*μ*m-wide fragment of hippocampal neuropil containing 54 excitatory synapses. Previous Monte Carlo models have dealt successfully with a comparable extent of neuropil surrounding one synapse (21, 26, 27, 28), including extracellular diffusion in a complex geometrical environment (29, 30). Here, we attempt simulations on a similar scale, but involving interactions of individual neurotransmitter molecules with scattered individual molecules of receptors. We simulate stochastic glutamate release from spatially separated synapses and analyze the occupancy dynamics of all receptors and transporters in the surrounding three-dimensional tissue. Our results predict that individual synaptic discharges are 10–30 times more efficient than nonsynaptic, slow glutamate release in providing sustained activation of NMDARs. Such activation could thus retain information about local excitatory activity in the network.

## Materials and Methods

### Receptor kinetics

The NMDAR kinetics was adopted from the earlier study (14) as follows:

$$2Glu+R\overset{k_{on1}}{\underset{k_{off1}}{⇌}}Glu+GluR\overset{k_{on2}}{\underset{k_{off2}}{⇌}}Glu_{2}R\begin{array}{c} \overset{\beta }{\underset{\alpha }{⇌}}Glu_{2}R*\\ \overset{k_{d-2}}{\underset{k_{d+2}}{⇌}}Glu_{2}RD \end{array},$$

where *R* denotes NMDA receptor, *Glu* is glutamate, *R*^∗^ is the open receptor, *Glu*_2_ indicates two glutamate molecules (bound to receptor), and *k*_on1_ = 10^6^ M^−1^ s^−1^, *k*_off1_ = 4.7 s^−1^, *k*_on2_ = 5 × 10^6^ M^−1^ s^−1^, *k*_off2_ = 9.4 s^−1^, *β* = 46.5 s^−1^, *α* = 91.6 s^−1^, *k*_*d*+2_ = 8.4 s^−1^, and *k*_*d−*1_ = 1.8 s^−1^. We have previously tested and verified the validity of this kinetic scheme against experimental NMDAR current recordings in various conditions and under various assumptions (23, 28, 31), including extended kinetic steps that account for Zn^2+^ sensitivity (32). Note that the second step in this reaction is substantially tighter than the first one, thus boosting the nonlinear relationship between ligand availability and receptor activation.

The simplified glutamate transporter (EAAT1/GLAST) schematic was adopted from previous studies (20, 23, 33) as follows:

$$\cdots \overset{k_{3}}{\to }Glu+T\overset{k_{+}}{\underset{k_{-}}{⇌}}GluT\overset{k_{2}}{\to }Glu_{in}+T_{trans}\overset{k_{3}}{\to }\cdots ,$$

where *Glu* denotes glutamate, *T* is the glutamate transporter, *Glu*_in_ is glutamate taken up, *T*_trans_ indicates the translocated (intracellular) transporter, and *k*_+_ = 5 × 10^6^ M^−1^ s^−1^, *k*_*−*_ = 100 s^−1^, *k*_2_ = 20 s^−1^, and *k*_3_ = 20 s^−1^ (recycling rate); note that the part on the left corresponds to glutamate outside, on the right represents the inside of the cell, and the ellipsis (…) denotes a loop.

In practice, we computed receptor kinetics using the standard stoichiometry matrix relationship (a representation of the system of linear differential equations),

$$dn_{j}/dt=S_{ji}\times f_{i},$$

in which *f*_*i*_ is the flux vector (the concentration of reactant or the products of concentration for reactants) for each of the *i*th reaction, and in matrix **S**_*ji*_, the *j*th row and the *i*th column represent the kinetic constants of the *i*th reaction and the *j*th resultants, respectively. The resulting *n*_*j*_ is the nonrepeating vector of *j*th compounds in a prearranged order. In Monte Carlo simulations, the algorithm governing random-walk glutamate diffusion and its binding with NMDARs was cross-validated by matching published outside-out patch recordings with the simulation outcome as described previously (23, 31).

### Monte Carlo model: microenvironment of individual synapses

Our simulation arena was based on one of the most common subjects of experimental investigations of neuronal plasticity and memory formation in the brain, area CA1 (stratum radiatum) of the hippocampus. The microenvironment of individual excitatory synapses formed by CA3 pyramidal axons on CA1 pyramidal cell dendritic spines in this area has been documented in detail (33, 34, 35, 36, 37) and successfully explored in several previous Monte Carlo models (21, 26, 38, 39). These include our earlier studies (23, 27) in which the core algorithms and their detailed verification against the published experimental recordings were outlined.

In brief, the presynaptic part (en-passant boutons) and the postsynaptic part (dendritic spine heads) of individual synaptic connections were represented by the face-to-face sides of two adjacent cubes separated by a 30-nm-high cleft (see Fig. 3 *A*). Cube sides were set at 470 nm, consistent with the typical dimensions of axonal boutons and dendritic spines in the hippocampal area CA1 (25, 34, 37). At each synapse, ∼20 NMDARs were scattered on the cube face representing the postsynaptic membrane, of which the central 250-nm-wide circle area was considered as the postsynaptic density containing synaptic NMDARs, in general agreement with the NMDAR immuno-labeling data obtained with transmission (16, 17) and freeze-fracture (40) electron microscopy of area CA1. It was also in line with the small number of NMDARs detected at individual dendritic spines in CA1 pyramidal cells using high-resolution Ca^2+^ imaging (41). This arrangement also followed the notion that the total numbers of NMDARs inside and outside synapses in this area are compatible (see Introduction). Glutamate transporters (EAAT1-2 type) were incorporated throughout the arena as an uptake reaction (see above) occurring on cube faces that were not the synaptic apposition zones.

The uptake intensity was equivalent to an average extracellular transporter density on the cell membrane surface of 5–10 × 10^3^ *μ*m^–2^, or the average extracellular volume density of ∼0.2 mM (42), and the kinetics as specified above. During synaptic discharge events, 3000 glutamate molecules were released into the center of the cleft (synaptic apposition faces), in agreement with experimental estimates (27, 43). The extracellular diffusion coefficient for glutamate was set at 0.4 *μ*m^2^/ms, which is between the intracleft value of ∼0.33 *μ*m^2^/ms estimated in electrophysiological experiments (44) and the estimated extracellular value due to viscosity, ∼0.45 *μ*m^2^/ms (23).

### Monte Carlo model: synaptic neuropil

Individual modeled synapses represented by cube faces (see Fig. 3 *A*) were combined into a regular cubic lattice to construct a Monte Carlo model of a neuropil fragment expanding to 3 × 3 × 3 *μ*m (see Fig. 3 *B*). With the typical synaptic volume density of ∼2 *μ*m^−3^ in this brain area (25), a total of 54 synaptic connections were thus uniformly randomly distributed on cube faces. Neighboring cubes were separated by a 30-nm extracellular gap, thus giving an extracellular space fraction *α* ∼0.17, in good correspondence with experimental measurements (45, 46, 47).

To reproduce a common experimental scenario, individual excitation pulses applied in accord with the chosen frequency generated synaptic discharges at individual release sites with a uniform probability across the sample. To maintain the unchanged amount of released glutamate at various frequencies we generated exactly six synaptic discharge release events per trial (for each frequency), thus engaging during the burst ∼11% of all synapses chosen randomly across the arena, as explained in the text. See Results for further detail and explanations relevant to specific simulation protocols.

### Computing environment: a dedicated PC cluster

Simulations were carried out using a dedicated, ad hoc-built 8-node BEOWULF-style diskless PC cluster running under the Gentoo LINUX operating system, an upgraded version of the cluster described in Zheng et al. (23). Each node contained a quad-core Intel Xeon processor and 4 GB of DDR3 RAM. All nodes are connected through a Gigabit Ethernet switch to a master computer that distributes programs and collects the results in its hard disk. The program was written and compiled in MATLAB and C++ (GNU C compiler). Initial tests with the cluster indicated that the technically feasible size of the modeled neuropil had to be restricted to a 3-*μ*m-wide cube, with 54 individual synapses, 1054 NMDARs, and ∼2 × 10^4^ individual glutamate molecules, and their reactions traced every 15 ns. A 100–200-ms run of the model took 3–6 days per trial of net computing time. Volume division has to be added on top of the original program (23) using the OCTREE algorithm, which divides the simulation volume into smaller self-contained units that communicate at their boundaries. Because parallelization was ideally suited for this purpose, computations involving Monte Carlo simulations were parallelized and optimized using routines and processed, as implemented by Sitrus LLC (formerly AMC Bridge LLC, Randolf, NJ).

## Results

### NMDAR activation: temporal summation in a series of release events

**Figure 1:**
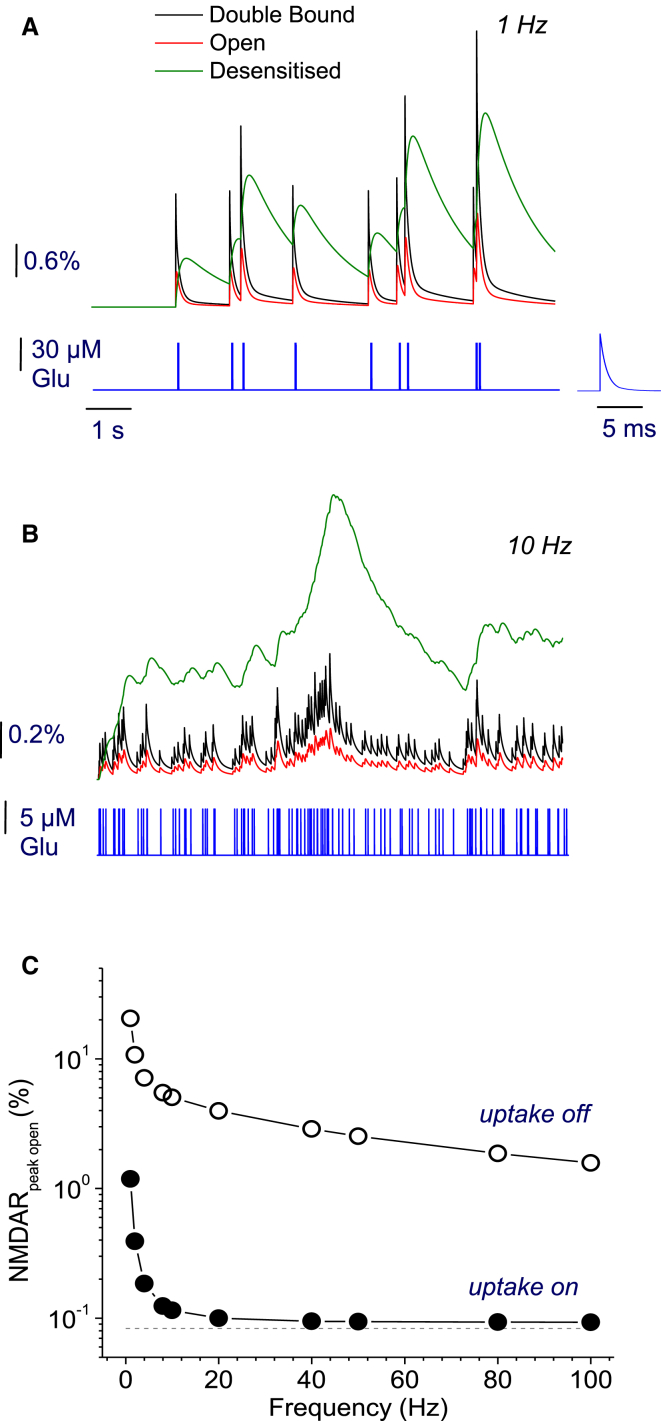
Temporal integration of NMDAR responses evoked by a sequence of glutamate release events. (*A* and *B*) Examples of NMDAR activation kinetics (*upper traces* in *A* and *B*) evoked by a series of brief glutamate release (*blue trace*; concentration time course during release magnified in *A*, *inset*) at two average event frequencies, 1 Hz (*A*) and 10 Hz (*B*). (*Black*, *red*, and *green traces*, respectively) Double-bound, open, and desensitized states of NMDARs. NMDAR kinetics were calculated using a multistep, single-compartment model (Materials and Methods). (*C*) Average occupancy of NMDARs at different release frequencies, with and without 0.2 mM glutamate transporters present (*solid* and *open circles*, respectively; NMDAR activation by glutamate assumes sufficient membrane depolarization to fully relieve the Mg^2+^ block). (*Dotted line*) Activation value under the ambient glutamate level equilibrated under steady-state glutamate supply (leakage) in the presence of transporters. The supply rate provides the amount of glutamate equivalent to that averaged over time during individual releases at various frequencies as shown. To see this figure in color, go online.

Because NMDARs have high affinity to glutamate, they could remain bound to the transmitter hundreds of milliseconds after a very brief (<1 ms) synaptic discharge. This enables NMDARs to be temporal integrators of glutamate release events. Although such a role for NMDARs has long been understood, its quantitative implications have not been explored. To get an insight into this integration in the time domain (without a spatial component), we first simulated responses of NMDARs to a random sequence of brief glutamate transients that mimic synaptic discharges. The mean frequency of such transients was varied between 1 and 100 Hz, to reflect the wide physiological range of principal cell firing in the brain. The waveform of individual glutamate pulses reproduced that in the vicinity of the synaptic cleft (20, 21, 28, 48). To compare the effect of individual release events with that of the ambient glutamate in a like-for-like fashion, simulated transients were scaled so that the overall, time-averaged extracellular glutamate concentration remained at ∼40 nM, similar to the background level measured in situ (4). This was achieved by appropriately scaling down the peak glutamate concentration (and therefore overall release) for individual events at higher frequencies (Fig. 1, *A* and *B*).

These simple kinetic computations have indicated that increasing the release frequency (while maintaining unchanged the total amount of glutamate released) monotonically reduces the average occupancy of NMDARs, asymptotically approaching the steady-state case equivalent to the effect of ambient glutamate (∼0.1% NMDAR activation with glutamate transporters present; Fig. 1 *C*, *dotted line*). In contrast, the average NMDAR occupancy elevates sharply at low release frequencies: at 1–2 Hz, NMDAR activation is >10-fold of that under steady-state exposure to 40–50 nM glutamate (Fig. 1). This change reflects nonlinearity of glutamate action on NMDARs, including the double-occupancy requirement, and thus a supralinear effect of increased glutamate concentrations on NMDAR activation. Furthermore, at ∼3–5 Hz (200–300 ms intervals), one could see a transition from individual transient NMDAR responses (normally lasting for 200–300 ms) to temporal summation leading to the overlapped receptor activation and inactivation time courses plus relative buildup of desensitization (compare Fig. 1, *A* and *B*).

In these tests, increasing pulse amplitudes at lower frequencies did not require an assumption of multivesicular release to justify our simulations: even at 1 Hz, the simulated concentration peak (∼0.12 mM) was still comparable with or less than, the average glutamate level inside the synaptic cleft over an ∼1-ms postrelease (20, 23, 27, 35, 49). In a functional context, concentration transients simulated here could be thought of as a crude approximation of the average exposure of NMDARs, which are predominantly near the activated synapses at lower frequencies (higher glutamate transients), and predominantly away from activated synapses at higher frequencies (lower glutamate transients).

### Two different aspects of the NMDAR kinetics contribute differently to synaptic signal integration

**Figure 2:**
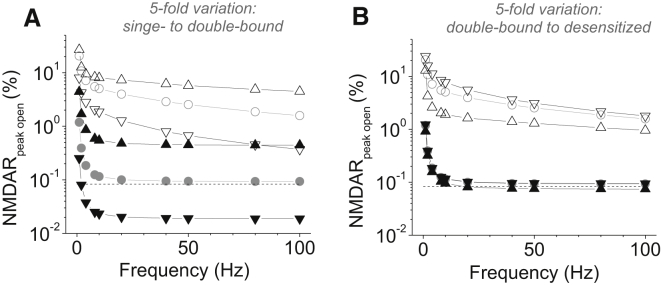
Double binding and desensitization have differential effects on the relationship between NMDAR activation levels and glutamate release frequency. (*A*) A further exploration of the model experiments shown in Fig. 1. Data depict average peak activation of NMDARs at different release frequencies, with 0.2 mM and 2 *μ*M glutamate transporters present (*solid* and *open signs*, respectively). (*Circles*), (*up triangles*), and (*down triangles*) correspond to the original model parameters, the fivefold-increased and fivefold-reduced rates, respectively, for the NMDAR transition from a single- to a double-bound state. Other conditions are as in Fig. 3. (*B*) Same symbol key as in (*A*), but with fivefold variation for the NMDAR transition from a double-bound to a desensitized state.

First, interestingly, NMDARs show higher desensitization levels relative to the occupancy or activation levels at higher frequencies (and lower amplitudes) of glutamate release (compare Fig. 1, *A* and *B*). As mentioned above, the latter scenario should be relevant to NMDARs reached by lower (and slower) glutamate waves outside active synapses. To understand better the contribution of receptor double-bound and desensitization states to its activation profile, we varied the corresponding NMDAR kinetic parameters by more than an order of magnitude (five-fold up and down) exploring the effect of transporters. Computations have revealed that, first, the single-bound to double-bound NMDAR transition rate has an increasingly strong influence on NMDAR activation with higher release frequencies (Fig. 2 *A*), whereas a transition from a double-bound to a desensitized state has a relatively modest effect (Fig. 2 *B*).

Second, glutamate uptake has an expectedly profound effect on NMDAR activation throughout the range of kinetic constants (Fig. 2). Previous studies reported that blockade of glutamate transport with DL-threo-*β*-benzylozyaspartic acid boosted 5–8-fold the standing NMDAR current in conditions of quiescent tissue (i.e., with no sustained synaptic activity) (3, 4). Our simulation results (Figs. 1 and 2) predict that during sustained synaptic activity transporter blockade should boost average NMDAR current by an order of magnitude or higher. This prediction seems fully consistent with the increased role of glutamate transport in such conditions compared to quiescent tissue. Overall, throughout the explored range of parameters, our data indicate that brief transients of glutamate appear much more efficient in generating average sustained NMDAR occupancy (at least in the time domain) than the steady-state exposure to the same total amount of glutamate released over the same time period.

### NMDAR activation: synaptic events in three-dimensional neuropil

Although the above analysis provides a basic understanding for temporal integration of release events by NMDARs, it neglects the complexities of the spatial aspect associated with glutamate diffusion in the brain. To evaluate the consequences of individual synaptic events in a realistic in situ environment, we developed a detailed three-dimensional Monte Carlo model of a neuropil fragment in which synaptic release sites, NMDARs, and glutamate transporters were distributed in accordance with the available experimental data.

**Figure 3:**
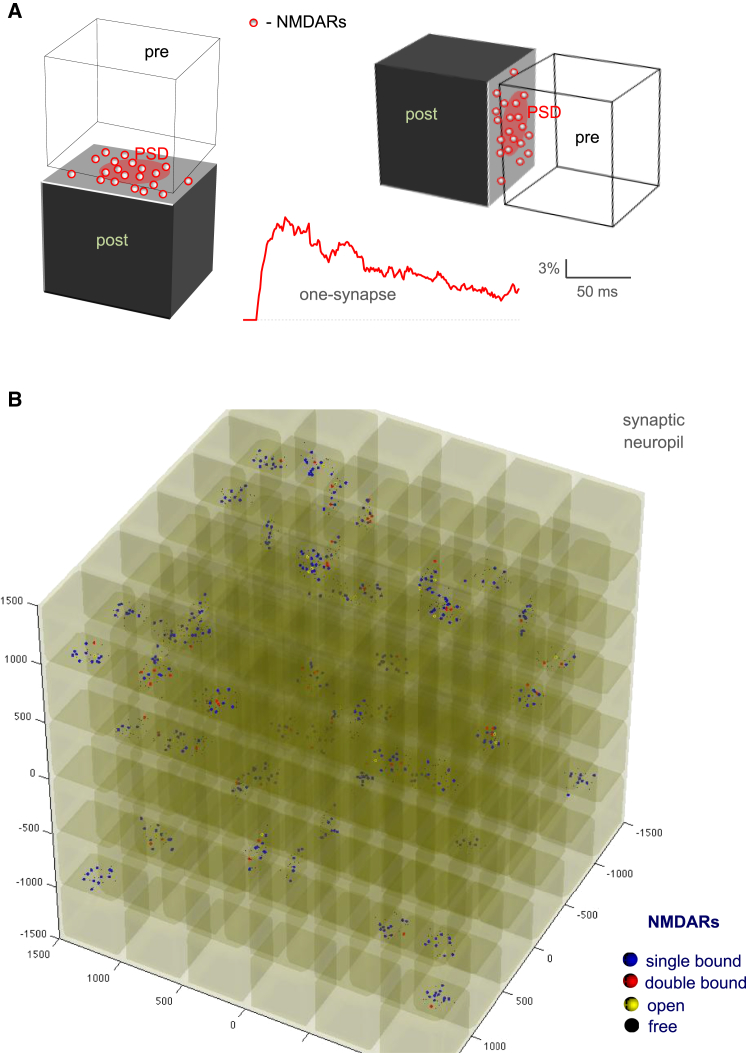
Activation of NMDARs during stochastic synaptic discharges in three-dimensional neuropils: a Monte Carlo model. (*A*) Pairs of adjacent cubes (side, 470 nm) representing simplified geometry of the typical excitatory (glutamatergic) synapse in the modeled hippocampal neuropil, with presynaptic and postsynaptic elements separated by the cleft, as indicated. NMDARs (*red dots*) are scattered inside and outside the postsynaptic density (*red oval*) on one side of the apposition area. High-affinity glutamate transport is enabled outside the apposition zone (*dark gray sides*) throughout the volume. (See Materials and Methods and Zheng et al. (23) for further detail, parameter exploration, and Monte Carlo validation tests.) (*Trace*) Typical NMDAR activation time-course at a single modeled synapse upon discharge of 3000 glutamate molecules into the cleft center. Monte Carlo simulation of individual receptor openings; *n* = 64 model runs. (*B*) A modeled fragment of synaptic neuropil (3 × 3 × 3 *μ*m cube) arranged as a regular cubic lattice (shown as a semi-translucent structure) that incorporates individual synaptic connections depicted in (*A*). (*Color-coded dots*) Snapshot of individual NMDARs 90 ms after cessation of six glutamate release events at 100 Hz. Colors indicate four different states, as shown; open receptors assume no Mg^2+^ block; in this example, glutamate uptake is disabled for simplicity. The extracellular volume fraction is ∼17%, the numerical synaptic density is 2 *μ*m^−3^ (54 synapses in total) in accord with experimental estimates. (See Materials and Methods for further detail and model parameters.) To see this figure in color, go online.

First, individual synaptic connections were modeled as pairs of pre- and postsynaptic elements represented by cuboids (Fig. 3 *A*). The key constraints and algorithms relevant to the environment of CA1 excitatory synapses in the context of this model were tested and validated in the previous studies (23, 27). Reassuringly, Monte Carlo simulations within this single-synapse environment in this context could reproduce NMDAR activation kinetics typical for experimental recordings (Fig. 3 *A*, *trace*).

Second, individual synaptic connections were put together, at an experimentally observed spatial density, to form a regular cubic lattice thus representing a three-dimensional 3-*μ*m-wide neuropil fragment (Fig. 3 *B*, Materials and Methods). Next, we simulated glutamate release events that represent individual synapses firing at frequencies between 1 and 50 Hz. In our sample of 54 synapses, single-synapse firing at 1 Hz thus corresponded to a total of 54 events per second, and firing at 50 Hz corresponded to 2700 events per second. To keep the total amount of released glutamate unchanged throughout trials (for comparison purposes, as above), we generated exactly six release events for each frequency, thus engaging ∼11% of all synapses in the burst. This number of release events produced the total amount of glutamate comparable with the volume-average 40–50 nM in the extracellular space (ambient level in quiescent tissue in situ (4)) maintained over the tested time-period. Because in the hippocampus one requires >4–5% of excitatory synapses discharging quasi-synchronously to fire a CA1 pyramidal cell (50), our model conditions appeared consistent with moderate to high synaptic activity in the local circuit. During each individual discharge, 3000 molecules of glutamate (27) were released instantaneously from a randomly selected release site. The average NMDAR occupancy level (which corresponds to the receptor open state in the absence of Mg^2+^ block) was computed using receptor state readout for all 1054 NMDARs during and after the burst.

### Integration of glutamate release events by NMDARs in three-dimensional neuropil

**Figure 4:**
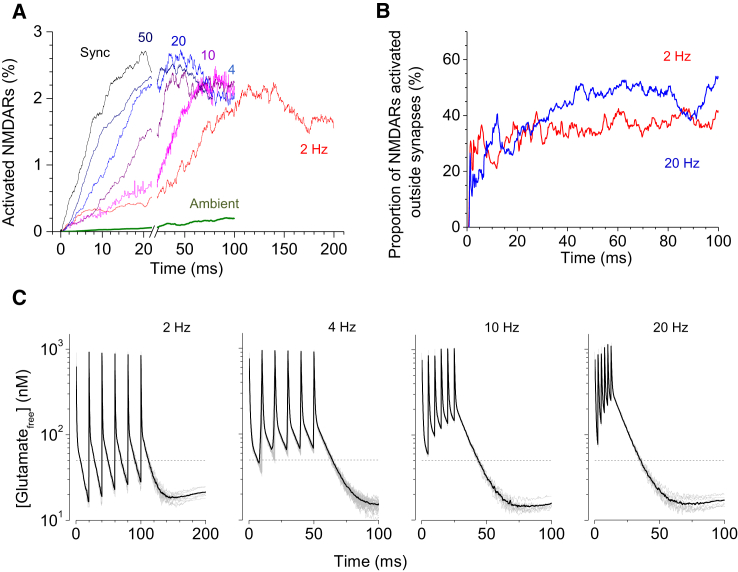
Spatial and temporal integration of NMDAR activation during glutamate release. (*A*) Time course of the NMDAR activation (open state with no Mg^2+^ block) resulting from six release events occurring at different frequencies within the area and, separately, with the equivalent amount of ambient (leaking) glutamate, as indicated; glutamate transporters are enabled at 0.2 mM extracellular concentration. The frequency values shown indicate events per the entire simulation arena: the frequency of events per synapse is therefore 54-times lower. Average of *n* = 8 runs (except for 4 Hz^∗^ with *n* = 64; this test frequency was chosen to test the outcome stability (53)). *Sync* is the synchronous release from six arbitrarily chosen synaptic sites. Traces at higher frequencies and for ambient glutamate are curtailed at 100 ms due to excessive demand for computing time and because the 2-Hz trace at 100–200 ms should be representative of other cases postrelease. (*B*) The proportion of activated NMDARs outside releasing (active) synapses at two characteristic frequencies, as indicated. Note the monotonic increase of the activation level reflecting the buildup of spatiotemporal glutamate signal integrating over the tissue volume. (*C*) Time course of the average concentration of free glutamate during and after six release events at different frequencies (within the area of 54 synapses), as indicated. (*Gray lines*) Individual runs (*n* = 8); (*black line*) average; and (*dotted gray line*) 50 nM (estimated ambient level of glutamate in situ). The rebound of glutamate concentration from its lowest value reflects unbinding of glutamate molecules from transporters after an initial transporter-glutamate buffering event, as described in Lehre and Rusakov (33). To see this figure in color, go online.

Simulation results with the three-dimensional neuropil indicate that, after the burst, the volume-average open-state NMDAR occupancy reaches 1.5–2.5%, lasting at this level for at least 200 ms (Fig. 4 *A*). This level is 20–30 times higher than the average 0.1% occupancy of the NMDAR open state generated by steady-state exposure to 40–50 nM glutamate. Reassuringly, a similar comparison arises when the ambient leakage of the same amount is introduced in the modeled neuropil in a non-steady-state fashion (*ambient trace* in Fig. 4 *A*).

Because the maximal activation level of NMDARs in the vicinity of release sites is ∼30%, the occupancy of 1.5–2.5% suggests that moderate synaptic activity in the brain could correspond to a standing NMDAR current that is only 10–20 times smaller than the postsynaptic NMDAR signal generated by all synaptic inputs to a given cell. Interestingly, NMDAR occupancy increases in a quasi-linear fashion with individual release events, with little dependence on the event frequency (including fully synchronized release). This indicates that the spatial overlap of glutamate clouds generated by distinct release sites does not reach concentration levels that prompt substantial supralinear activation of NMDARs (similar to that illustrated in Fig. 1 *B* for lower frequencies). Instead, the sustained activation level of NMDARs appears to reflect the total number, rather than the current rate, per se, of release events. Intriguingly, our simulations suggest that 30–40% of activated NMDARs actually occur outside activated synapses (Fig. 4 *B*). This phenomenon is consistent with earlier estimates in this brain area obtained in electrophysiological experiments (50, 51, 52, 53), thus pointing to the extent of extrasynaptic glutamate actions.

Finally, our three-dimensional model also predicts that the average concentration of free extracellular glutamate shortly (10–50 ms) after release events falls back to the level that is lower than or comparable to the ambient glutamate level in quiescent tissue (Fig. 3 *C*). These results suggest that synaptic discharges, while leading to substantial sustained occupancy of NMDARs, do not generate the background glutamate concentration above what is detected in an effectively silent neuropil.

## Discussion

The main findings of this study are as follows. First, our simulations predict that moderate activity of sparsely distributed excitatory synapses is sufficient to provide substantial sustained open-state occupancy of NMDARs in three-dimensional brain tissue. Perhaps the key aspect of spatiotemporal signal integration in this context is that individual glutamate molecules which escape from individual synapses and singly-bind to some extrasynaptic NMDARs remain receptor-bound for >100 ms. Thus individual activated synapses leave tagged areas for NMDAR activation upon subsequent glutamate releases nearby. It appears that such synaptic activity can readily lead to the ∼2% NMDAR occupancy level, which is 10–30 times higher than the occupancy produced by the nonsynaptic background supply, or leakage, of the comparable amount of glutamate. Because glutamate release events are orders-of-magnitude shorter than the NMDAR activation timescale (submillisecond versus 100–300 ms), NMDARs play the role of an efficient temporal integrator for rapid glutamate signals. Intriguingly, within the tested range of synaptic activity the receptor occupancy depends on the number, rather than frequency, of release events. This near-linear integration occurs mainly because the probability for two events to occur in the same three-dimensional neighborhood is relatively low unless the neighboring inputs are purposefully synchronized or unless the proportion of active synapses is much higher than the proportion sufficient to fire the target pyramidal cell (4–5%).

Second, our simulations predict that sparse synaptic discharges resulting in relatively high standing occupancy of NMDARs generate a background (ambient) level of glutamate unlikely to exceed that in quiescent tissue. These data also generate some testable predictions: for instance, blockade or facilitation of vesicular release should not exert major effects on ambient glutamate while altering the average NMDAR activation in situ accordingly.

There are some potentially important implications of these observations: First, they suggest that excitatory network activity can regulate the average NMDAR occupancy without significant rises in the background glutamate level. This implies that measurements of the ambient glutamate in active brain tissue are unlikely to provide insights into the extent of sustained NMDAR activation or excitatory synaptic activity. More likely, such measurements will report the state of glutamate uptake or removal. Indeed, in functional terms, keeping the ambient glutamate low is critical for enabling a high signal/noise when activating high-affinity synaptic glutamate receptors, both ionotropic and metabotropic, by rapid synaptic discharges. The low background level of glutamate is also important to minimize prolonged desensitization of glutamate receptors. Second, our data provide further support to the argument that a significant proportion (30–40%) of NMDARs activated by synaptic activity are likely to occur outside active synapses. Third, and finally, the outcome of simulations suggests that, by integrating synaptic signals in space and time, glutamate-occupied NMDARs can accumulate and retain information about the average activity of local excitatory circuits. At any given time, the NMDAR occupancy level could translate this information into a cellular signal, such as local Ca^2+^ entry, once the NMDAR Mg^2+^ block is removed. For the latter to happen, the target dendrite has to be depolarized, for instance, by a back-propagating action potential, as was reported in 2012 (24). Thus, the mechanism combining NMDAR occupancy and dendritic depolarization could enable individual neurons to obtain a precisely-timed functional readout of how much local excitatory activity has recently occurred.

## Author Contributions

D.A.R. and K.Z. designed the study; K.Z. set up the computational environment, built the model, and carried out simulations; and D.A.R. and K.Z. wrote the article.
